# Value of white matter hyperintensity volume and total white matter volume for evaluating cognitive impairment in patients with cerebral small-vessel disease

**DOI:** 10.3389/fnagi.2023.1096808

**Published:** 2023-03-31

**Authors:** Sen Zhang, Yaya Hu, Huilin Yang, Qianqian Li, Jing Chen, Hongying Bai

**Affiliations:** Department of Neurology, The Second Affiliated Hospital of Zhengzhou University, Zhengzhou, China

**Keywords:** white matter hyperintensity, white matter volume, cerebral small-vessel disease (CSVD), brain atrophy, cognitive impairment, cognitive dysfunction, risk factors, aging

## Abstract

**Background:**

White matter hyperintensities (WMH) are a key imaging feature of cerebral small-vessel disease (CSVD). However, there is a lack of standardized methods for determining WMH volume, and the value of total white matter (WM) volume in the assessment of cognitive impairment in patients with CSVD remains unknown.

**Objective:**

We aimed to explore the correlations of WMH volume and WM volume with cognitive dysfunction and its components in patients with CSVD. We also aimed to compare the value of the Fazekas score, WMH volume, and ratio of WMH volume to total WM volume in the assessment of cognitive dysfunction.

**Methods:**

The study included 99 patients with CSVD. Patients were categorized into following groups based on MoCA scores: patients with mild cognitive impairment and those without. Brain magnetic resonance images were processed to investigate differences in WMH and WM volumes between the groups. Logistic regression analysis was used to determine whether these two factors were independent risk factors for cognitive dysfunction. Correlation analysis was used to examine the relationships of WMH and WM volume with different types of cognitive impairment. Receiver operating characteristic curves were used to compare the effectiveness of the WMH score, WMH volume, and WMH to WM ratio for evaluating cognitive dysfunction.

**Results:**

There were significant differences in age, education level, WMH volume, and WM volume between the groups (*P* < 0.05). After adjusting for age and education, the multivariate logistic analysis indicated that both WMH volume and WM volume were independent risk factors for cognitive dysfunction. Correlation analysis indicated that WMH volume was mainly related to cognition involving the visual space and delayed recall. WM volume was not strongly associated with different types of cognitive dysfunction. The WMH to WM ratio was the strongest predictor, with an area under the curve value of 0.800 and a 95% confidence interval of 0.710–0.891.

**Conclusion:**

Increases in WMH volume may aggravate cognitive dysfunction in patients with CSVD, and a higher WM volume may reduce the effect of WMH volume on cognitive function to a certain extent. The ratio of WMH to total WM volume may reduce the impact of brain atrophy, allowing for more accurate evaluation of cognitive dysfunction in older adults with CSVD.

## 1. Introduction

Cerebral small-vessel disease (CSVD), the most common form of chronic progressive vascular disease, is characterized by decreases in the size of the arteries, capillaries, and veins that supply the white matter (WM) and deep structures of the brain. The incidence of CSVD is very high, particularly among older adults, who are at risk of complications including stroke, gait disorders, mental/cognitive disorders, urinary disorders, and other major events (Pantoni, 2010; Ter Telgte et al., 2018; Cannistraro et al., 2019).

Magnetic resonance imaging (MRI) plays a key role in the diagnosis of CSVD. The imaging features of CSVD can be categorized as white matter hyperintensities (WMH), lacunar infarction, cerebral microbleeds, enlarged perivascular space, brain atrophy, and others (Li et al., 2018; Chen et al., 2019). One previous study reported that baseline WMH increased the risk of cognitive impairment and all-cause dementia (ACD) by 14%. WMH also increases the risks of Alzheimer’s disease and vascular dementia by 25 and 73%, respectively. High-grade WMH and consistently increased volume or severity of WMH have been shown to increase the risk of dementia (Hu et al., 2021).

At present, methods for evaluating WMH mainly include the Fazekas scale (Fazekas et al., 1987) and professional image processing software, which can be used to extract the volumes of periventricular WMH, deep WMH, and WM. Some studies have shown that the pathogenesis of periventricular WMH differs from that of deep WMH. For example, cognitive impairment has been associated with lateral ventricular WMH but not deep WMH (de Groot et al., 2000). However, DeCarli et al. (2005) reported a high correlation between the WMH volumes, suggesting that it is not necessary to distinguish between them. A major factor in this discrepancy is the lack of standardized methods for determining WMH volume, which prevents comparison of data among healthy adults included in different studies (Melazzini et al., 2021). A study of Alzheimer’s disease demonstrated that WMH and atrophy of lobes and gray matter are related to cognition (Ramusino et al., 2022). However, differences in WM volume among different patients are rarely considered in studies of WMH and cognitive dysfunction, highlighting the need to address this issue to advance clinical research. Therefore, in the present study, we explored the relationships among WMH volume, WM volume, and cognitive function in hospitalized patients with CSVD. We further aimed to determine whether the ratio of WMH to total WM volume can better assess cognitive impairment than WMH. There are two hypotheses about cognitive dysfunction caused by WM volume: 1. There are very complex nerve conduction bundles in the WM, and the reduction of their volume may affect or destroy this nerve fiber network, thus affecting cognitive function. 2. WMH are areas of the degeneration of normal WM structure, which plays a role in promoting cognitive impairment in the elderly. We evaluated whether the larger WM volume represents a stronger compensatory ability after the generation of WMH. If this compensatory ability exists, the assessment of WMH volume may need to be corrected according to the WM volume.

## 2. Materials and methods

### 2.1. Participants

The study included 99 patients with CSVD who were hospitalized in the Department of Neurology at the Second Affiliated Hospital of Zhengzhou University and Xinzheng Public People’s Hospital between January 2021 and August 2022. Inclusion criteria were as follows: (a) meeting the neuroimaging diagnostic criteria for CSVD (Wardlaw et al., 2013); (b) absence of mental or neurological disorders, sufficient ability to communicate, and ability to cooperate with treatment; (c) age ≥18 years, (d) provision of written informed consent; and (e) availability of complete clinical data. Exclusion criteria were as follows: (a) secondary WM lesions; (b) secondary diseases that can cause cognitive dysfunction, such as stroke and Alzheimer’s disease; (c) large MRI artifacts making it difficult to measure volumes; and (d) serious systemic diseases, such as severe systemic infection, disseminated intravascular coagulation, and cardiopulmonary failure. The study was approved by the Ethics Committee of the Second Affiliated Hospital of Zhengzhou University. The patients provided their written informed consent to participate in this study.

### 2.2. Data collection and assessment methods

The following baseline data were collected for all patients: age, sex, education level, history of hypertension, diabetes, coronary heart disease, and other general information. Digital imaging and communications in medicine (DICOM) data for cranial MRIs were obtained for each participant. All participants underwent evaluation using the Montreal Cognitive Assessment (MoCA) (Nasreddine et al., 2005). MoCA scores were corrected according to the level of education, with 1 point added to the total score for those with less than 12 years of education. The Fazekas scale scored the periventricular and deep WMH separately. Periventricular WMH: 0, no lesion; 1, cap-like or pencil-like thin layer signal near the lateral ventricle; 2, the lesion was a smooth halo; 3, irregular high signal near the ventricle and extended to the deep WM. Deep WMH: 0, no lesion; 1, punctate lesions; 2, a small part of the lesion fusion; 3, large area of lesion fusion. The total score for WMH was obtained by summing the two (Fazekas et al., 1993).

### 2.3. Imaging

Magnetic resonance imaging was performed using a Siemens MAGNETOM Skyra 3.0T MRI scanner. The examination sequences included T1-weighted images, T2-weighted images, T2 fluid-attenuated inversion recovery (FLAIR) images, and magnetic resonance angiography (MRA). The field-of-view (FOV) of the T1-weighted images was 260 mm × 260 mm, the matrix was 160 × 160, the slice thickness was 1.6 mm, and there were 128 sagittal images. The FOV of the T2-weighted images was 240 mm × 240 mm, the matrix was 384 × 384, the slice thickness was 5 mm, and there were 20 axial images. The FOV of the T2 FLAIR images was 215 mm × 230 mm, the matrix was 360 × 384, the scanning layer thickness was 5 mm, and there were 20 horizontal images.

### 2.4. Volume measurement

**FIGURE 1:**
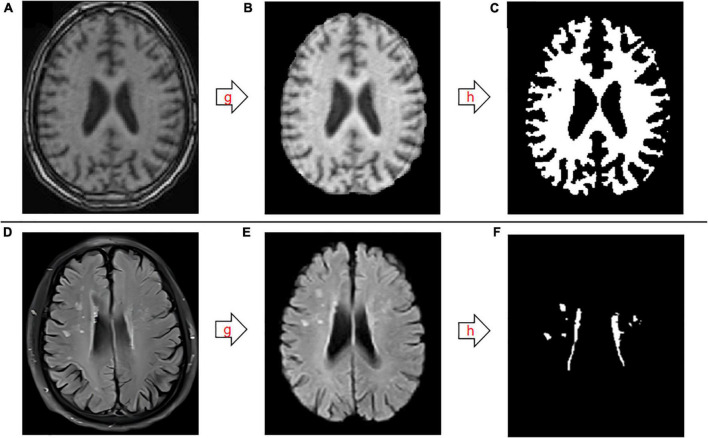
Schematic diagram of the segmentation and extraction process for white matter and white matter hyperintensities. **(A–C)** T1-weighted images. **(D–F)** T2-weighted fluid-attenuated inversion recovery images. g, skull stripping; h, threshold segmentation.

The imaging files were processed using the professional medical image FMRIB Software Library (FSL) v6.0. FSL was developed by the Brain Functional Magnetic Resonance Imaging Center (FMRIB) of Oxford University in the UK. FSL can process structural and functional nuclear magnetic resonance using a computer to accurately extract lesions and segment brain tissue structures (Jenkinson et al., 2012). T1- and T2 FLAIR images were processed successively by stripping the skull, followed by threshold segmentation using an algorithm that divides the gray level of the image into different grades and determines the boundary of the object to be segmented by setting a gray threshold (Figure 1). The specific operation process was as follows: first, we entered the decompression image directory, and then used bet for segmentation. The volume of the image of the operation is the volume of intracranial volume. Next, the software tissue type segmentation FAST was used to segment the brain tissues of different structures, namely GM, WM, and CSF. The segmented image was imported into 3D-slicer software (Gonzalo Domínguez et al., 2016), and a segment was added through the Add button in the software. The image was modified, 3D reconstructed, and the volume was measured using the following commands: None, Draw, and Erase, combined with the brain tissue anatomy. Normalized WM volume was the ratio of WM volume to intracranial volume.

### 2.5. Statistical analysis

Statistical analyses were performed using SPSS version 26.0. The Kolmogorov–Smirnov test was used to test the normality of quantitative data. Quantitative data conforming to a normal distribution were expressed as (x ± s), and those not conforming to a normal distribution were expressed as *M* (P25, P75), “x ± s” is the “mean ± standard deviation” and “*M* (P25, P75)” is the median (25th percentile, 75th percentile). Count data were expressed as relative numbers. An independent samples *t*-test was used for inter-group analysis of normally distributed data, and the rank sum test was used for inter-group analysis of non-normally distributed data. Multivariate logistic regression analysis was used to explore risk factors such as cognition and exercise. Spearman rank correlation analysis was used to analyze the correlations between non-normally distributed variables, with |*r*| < 0.4 indicating weak correlation; 0.4 ≤ |*r*| < 0.7 indicating moderate correlation, and 0.7 ≤ | *r*| < 1 indicating strong correlation. FDR correction of *P*-values in multiple testing using the Benjamini and Hochberg method. FDR (*q*) < 0.05 was considered meaningful. Receiver operating characteristic curve (ROC) analysis was used to analyze the influence of the Fazekas score, volume of WMH, WM volume, and the ratio of WMH volume to WM volume on cognitive dysfunction in patients with CSVD. AUC values of 0.5–0.7, 0.7–0.9, and ≥0.9 were considered to indicate low, moderate, and high accuracy, respectively. *P*-values < 0.05 were considered statistically significant.

## 3. Results

### 3.1. General data

A total of 99 patients with CSVD who met the inclusion criteria were included in the study. Age and WMH volume were not normally distributed (*P* < 0.05) while WM volume (*P* = 0.14) and intracranial volume (*P* = 0.2) were. Participant average age was 70 (63, 73) years, and there were 61 men and 38 women. Table 1 shows the detailed characteristics of the included patients.

**TABLE 1:** Baseline characteristics.

Characteristics	*n* (%), x ± s or *M* (P25, P75) (*n* = 99)
Male	61 (61.6%)
Age [*M* (P25, P75), years]	70 (63, 73)
Hypertension	61 (61.6%)
Diabetes	26 (26.3%)
Coronary heart disease	28 (28.3%)
Education level [*M* (P25, P75), years]	9 (6, 12)
WMH volume [*M* (P25, P75), cm^3^]	9.89 (4.77, 16.36)
WM volume (x ± s, cm^3^)	499.84 ± 34.77
Intracranial volume (x ± s, cm^3^)	1,463.43 ± 61.12
MoCA score [*M* (P25, P75), points]	23 (20, 27)

WMH, white matter hyperintensity; WM, white matter; MoCA, Montreal cognitive assessment.

### 3.2. Correlation of WM volume and WMH volume with different degrees of cognitive impairment

The patients were divided into two groups: a mild cognitive impairment (MCI) group (*n* = 65) including patients with MoCA scores <26 and a non-MCI group including patients with MoCA scores ≥26. WM volume [*P* (MCI) = 0.2, *P* (non-MCI) = 0.2], intracranial volume [*P* (MCI) = 0.06, *P* (non-MCI) = 0.2], and normalized WM volume [*P* (MCI) = 0.2, *P* (non-MCI) = 0.2] of two were normally distributed. There were significant differences in age, education level, WMH, and WM volume between the groups (*P* < 0.05). As shown in Table 2, there were no significant differences in sex, hypertension, diabetes, coronary heart disease, or intracranial volume between the two groups (*P* > 0.05).

**TABLE 2:** Comparison of baseline data between the MCI and non-MCI groups.

Items	MCI group (*n* = 65)	Non-MCI group (*n* = 34)	*X*^2^/*Z*/*t*	*P*
Male (*n*, %)	40 (61.54)	21 (61.76)	0.000	0.982
Age [*M* (P25, P75), years]	71 (64, 74)	65 (56, 72)	2.129	0.033
Hypertension (*n*, %)	39 (60)	22 (64.70)	0.209	0.648
Diabetes (*n*, %)	18 (27.69)	8 (23.53)	0.200	0.655
Coronary heart disease (*n*, %)	19 (29.23)	9 (26.47)	0.084	0.772
Education level [*M* (P25, P75), years]	8 (4, 12)	11 (8, 14)	-2.641	0.008
WMH volume [*M* (P25, P75), cm^3^]	12.11 (6.97, 21.01)	4.61 (2.48, 11.31)	4.657	<0.001
WM volume (x ± s, cm^3^)	491.97 ± 30.33	514.87 ± 38.09	3.038	0.004
Intracranial volume (x ± s, cm^3^)	1,458.23 ± 51.21	1,473.36 ± 76.51	-1.038	0.305
Normalized WM volume (x ± s, %)	33.75 ± 1.95	34.99 ± 2.49	-2.715	0.008

MCI, mild cognitive impairment.

### 3.3. Multivariable logistic analysis of factors predicting cognitive impairment

Multivariable logistic regression analysis was performed to determine risk factors for cognitive impairment. Age, educational level, WMH, WM volume, and WM normalized volume were included. The stepwise forward method was used to identify WMH (*P* = 0.001, OR = 1.195, 95% CI: 1.074, 1.330), WM volume (*P* = 0.003, OR = 0.976, 95% CI: 0.960, 0.992), and education level (*P* = 0.039, OR = 0.875, 95% CI: 0.771, 0.993) as predictors of cognitive impairment; no collinearity was observed between them. The correlation analyses indicated that patients with high WMH volume, low WM volume, and low education level were more likely to develop MCI (Table 3).

**TABLE 3:** Multivariable logistic analysis of factors predicting cognitive impairment.

Variable	*b*	SE	Wald χ^2^	*P*	OR (95% CI)
Constant	12.453	4.168	8.928	0.003	–
WMH volume	0.178	0.055	10.652	0.001	1.195 (1.074, 1.330)
WM volume	-0.024	0.008	8.999	0.003	0.976 (0.960, 0.992)
Education level	-0.133	0.065	4.249	0.039	0.875 (0.771, 0.993)

WMH, white matter hyperintensity; WM, white matter; CI, confidence interval; *b*, regression coefficient; SE, standard error; Wald χ^2^, Chi-square value; OR, odds ratio.

### 3.4. Spearman rank correlation analysis between WMH volume, WM volume, and different cognitive components

The MoCA contains domains related to visual space and execution, naming, attention, speech, abstraction, delayed memory, and orientation ability. WMH volume exhibited moderate correlations with the total MoCA score and with subscale scores for visual space and execution and delayed memory (*P* < 0.05, *q* < 0.05). Total WM volume was weakly correlated with subscale scores for visual space and execution, attention, language, and delayed memory (*P* < 0.05, *q* < 0.05) (Table 4). Partial correlation analysis revealed that age and education level were significantly correlated with WMH volume (*P* < 0.05, *q* < 0.05), but not with WM volume (*P* > 0.05, *q* > 0.05). We used Spearman partial correlation analysis with age and education level as control variables and found that WMH volume was weakly correlated with the total MoCA score and with subscale scores for visual space and execution, delayed memory, orientation, visual space and execution, and attention (*P* < 0.05, *q* < 0.05). WM volume was moderately correlated with MOCA total score and subscale scores for attention. WM volume was weakly correlated with subscale scores for visual space and execution, orientation, language, and delayed memory (*P* < 0.05, *q* < 0.05) (Table 5).

**TABLE 4:** Results of the correlation analysis.

	WMH volume			WM volume		
	***r*_s_**	***P***	***q***	***r*_s_**	***P***	***q***
MoCA score	-0.531	<0.001	<0.001	0.393	<0.001	<0.001
Visual space and execution	-0.442	<0.001	<0.001	0.322	0.001	0.003
Naming	-0.192	0.056	0.056	0.113	0.266	0.333
Attention	-0.246	0.014	0.018	0.362	<0.001	<0.001
Language	-0.297	0.003	0.005	0.343	0.001	0.003
Abstract	-0.241	0.016	0.018	0.150	0.139	0.199
Delayed memory	-0.442	<0.001	<0.001	0.251	0.012	0.020
Orientation	-0.361	<0.001	<0.001	0.350	<0.001	<0.001
Age	0.418	<0.001	<0.001	-0.025	0.809	0.865
Education level	-0.285	0.004	0.006	-0.029	0.778	0.865

WMH, white matter hyperintensity; WM, white matter; *r*_s_, Spearman rank correlation coefficient; *q*, false discovery rate (FDR).

**TABLE 5:** Results of the partial correlation analysis.

	WMH volume			WM volume		
	***r*_s_**	***P***	***q***	***r*_s_**	***P***	***q***
MoCA score	-0.336	0.001	0.008	0.458	<0.001	<0.001
Visual space and execution	-0.224	0.016	0.032	0.385	<0.001	<0.001
Naming	-0.018	0.863	0.863	0.097	0.343	0.343
Attention	-0.232	0.022	0.035	0.413	<0.001	<0.001
Language	-0.204	0.045	0.060	0.324	0.001	0.002
Abstract	-0.112	0.277	0.317	0.154	0.132	0.151
Delayed memory	-0.307	0.002	0.008	0.249	0.014	0.019
Orientation	-0.285	0.005	0.013	0.348	<0.001	<0.001

Controlled variables: age, education level. WMH, white matter hyperintensity; WM, white matter; *r*_s_, Spearman rank correlation coefficient; *q*, false discovery rate (FDR).

### 3.5. Receiver operating characteristic curve analysis

**FIGURE 2:**
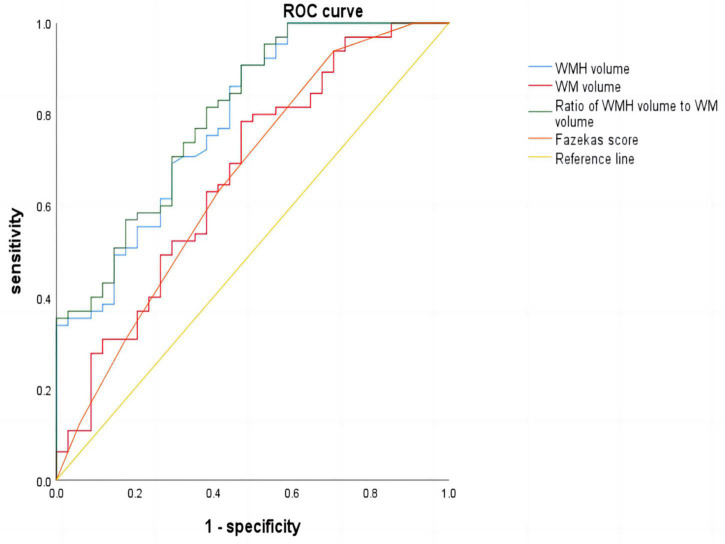
Receiver operating characteristic (ROC) curve.

Receiver operating characteristic curve analysis was used to analyze the influence of the Fazekas score, WMH volume, WM volume, and the ratio of WMH volume to WM volume on cognitive dysfunction in patients with CSVD. The ratio was the strongest predictor, with an area under the curve (AUC) value of 0.800 and a 95% CI of 0.710–0.891. When the threshold was 0.97%, the sensitivity and specificity for identifying cognitive dysfunction in patients with CSVD were 0.908 and 0.529, respectively, yielding the largest Youden index. The second highest AUC was observed for WMH volume (AUC = 0.786), with a 95% CI of 0.692–0.880. When the threshold was 4.72, the sensitivity and specificity for identifying cognitive impairment in patients with CSVD were 0.908 and 0.529, respectively, yielding the largest Youden index (Figure 2 and Table 6).

**TABLE 6:** Receiver operating characteristic (ROC) curve parameters.

Index	AUC	Cut-off value	Youden index	Sensitivity	Specificity
WMH volume	0.786	4.72	0.437	0.908	0.529
WM volume	0.667	511.335	0.314	0.785	0.471
Ratio	0.800	0.966	0.437	0.908	0.529
Fazekas score	0.658	-	0.232	0.938	0.294

AUC, area under the curve; WMH, white matter hyperintensity; WM, white matter.

## 4. Discussion

Previous studies have reported associations of CSVD with cognitive impairment, mental disorders, gait disorders, urinary incontinence, and other problems that represent risk factors for a variety of acute and chronic neurological diseases in the older adult population (Pantoni, 2010; Ter Telgte et al., 2018; Cannistraro et al., 2019; Chen et al., 2019; Hu et al., 2021). WMH is a key imaging feature of CSVD and has also been associated with cognitive dysfunction, stroke, Alzheimer’s disease, dementia, and death. WMH may also lead to cognitive dysfunction via secondary demyelination and subsequent neuronal loss (Sachdev et al., 2004; Wang et al., 2021).

At present, the assessment of WMH severity in CSVD is mainly based on a simple qualitative analysis of the Fazekas score (Fazekas et al., 1987). This scoring method can be used to assess the severity of WMH to a certain extent and has exhibited associations with cognitive dysfunction. However, in clinical work, researchers have gradually observed that scores may be the same for WMH manifestations with significant differences in severity, meaning that it may not accurately reflect the extent of cognitive dysfunction in these patients.

Advanced computational and imaging methods play an increasingly important role in medical diagnostics, and the capacity for quantitative analysis of brain tissue has gradually matured over the last several decades. Quantitative studies have reported that high WMH volume increases the risk of cognitive impairment and movement disorders in older adults (Su et al., 2017; Hu et al., 2021). Other studies have indicated that increased WMH volume is associated with more severe affective symptoms in patients with bipolar I disorder, suggesting a link between WMH and mental symptoms (Tighe et al., 2012; Tully et al., 2020). One study that analyzed WMH volume and gray matter volume reported a negative correlation between the two, noting that the relationship between WMH volume and cognitive impairment was independent of total gray matter volume (Boyle et al., 2016). Another study, which demonstrated a correlation between WMH and both cognitive and motor dysfunction, reported a correlation between the progression of WMH and the volume of cerebrospinal fluid in the lateral ventricle (Silbert et al., 2008). A study using machine learning (ML) combined with magnetic resonance imaging (MRI) demonstrated that the microstructure of WM is associated with different types of dementia (Palesi et al., 2018; Castellazzi et al., 2020). It proves the basis of the influence of WM structure on cognitive impairment.

In the current study, we analyzed relationships among WMH volume, WM volume, and cognitive dysfunction in patients with CSVD. Significant differences in WMH and WM volumes were observed between patients with and without MCI. Previous studies have demonstrated that age and education level have an impact on cognitive function in patients with CSVD (Pinter et al., 2015), consistent with our findings. A multivariate analysis incorporating WMH volume, WM volume, age, and education level identified both WMH volume and WM volume as independent risk factors for cognitive impairment. A previous meta-analysis demonstrated that WMH volume was associated with the risk of cognitive impairment and it supported our findings (Guo and Shi, 2022). A study on subclinical cerebral small vessel disease and processing speed in non-dementia patients (Hotz et al., 2021) revealed a correlation between WMH volume, normal WM volume and processing speed, also demonstrating that education level was a protective factor for WM volume and normal WM volume. WMH volume was also found to be associated with education and cognitive impairment (Xia et al., 2020). This is consistent with our current observation that WM volume and WMH volume were risk factors for cognitive impairment, and WMH volume was inversely proportional to education level. However, the total WM volume we used did not show a relationship with education level. Another study also demonstrated that WMH volume is associated with mild impairment of processing speed and executive function (Nylander et al., 2018). This supports our finding that the WMH volume is highly correlated with the visual space and execution scores in the MOCA scale.

The innovation of this research is reflected in the following aspects. First, when exploring the relationship between WM high signal volume and cognitive impairment, we included WM volume as a variable. We found that both WM high signal volume and WM volume were associated with cognitive impairment. The correlation was still observed after adjusting for age, education and other related factors. Secondly, correlation analyses suggested that there are differences in the correlation between these WM high signal and WM volumes and different types of cognitive impairment. This finding demonstrated that the two variables affect cognitive function through different underlying mechanisms which may be related to pathology. Thirdly, we observed that the absolute WM volume was more closely related to cognitive function than the normalized volume corrected by intracranial volume. WMH volume was related to age and education level, and the correlation with cognitive dysfunction was reduced after controlling for these variables. However, WM volume was not related to age or education level, and the correlation with cognitive impairment was only slightly affected by these variables. Further, we used the ratio of WMH volume to WM volume as an index to evaluate cognitive impairment and found that this index was superior to WMH volume in evaluating and predicting cognitive impairment.

Postmortem MRI studies have shown that WMH-related pathologies include mild microglial activation, astrocyte proliferation, oligodendrocyte reduction, myelin or axon loss, and perivascular space dilation (Fazekas et al., 1991). Astrocytes are the most abundant glial cells in the central nervous system (CNS). Although they were long regarded as passive cells that provide structural support for neurons, astrocytes are now thought to play a necessary role in supporting and maintaining a healthy CNS. There are two main subtypes of astrocytes: protoplasmic and fibrous astrocytes, which are located in the gray matter and WM (Lebel and Deoni, 2018). Glutamate, a major excitatory neurotransmitter in the human CNS, plays a role in a variety of neurological diseases (Beart and O’Shea, 2007) and is involved in communication between axons and glial cells through synaptic release deep in the WM (Alix and Domingues, 2011). Destruction of the synaptic structure between astrocytes or their axons may be the pathological basis by which WM lesions induce cognitive dysfunction.

Notably, many astrocyte lesions manifest as frontal lobe dominance (van der Knaap and Bugiani, 2017), indicating that astrocytes in the frontal WM and/or their synaptic structures may be more fragile than other WM regions and are more affected by WMH and decreases in WM volume. A study of the nerve fiber bundles in the WM of the frontal lobe demonstrated that hand kinematics and visual motion processing are related to the anatomical structure of the WM network in the frontal lobe (Budisavljevic et al., 2017), in accordance with the strong correlation observed between WMH and visual space and executive function in the current study (based on MoCA scores). WM lesions can induce a variety of disconnection syndromes, such as conduction aphasia, associative visual agnosia, apraxia, and pure alexia (Catani and ffytche, 2005), further supporting the notion that WMH and WM volumes are related to the pathological basis of cognitive dysfunction.

In the current study, the correlation between WMH volume and delayed memory was relatively strong, while correlations with visual, attention, orientation, verbal, and other aspects were weak. However, the specific reasons for this difference remain unclear. Correlations between total WM volume and different types of cognitive dysfunction were also weak. Although total WM volume may have a more comprehensive impact on different types of cognitive dysfunction, strong individual differences in compensatory ability may limit its value for predicting changes in cognitive function. However, our results indicated that the ratio of WMH to WM volume was most effective in identifying cognitive function in our patients with CSVD. This ratio may correct for the effect of brain atrophy or individual differences in WMH volume to a certain extent. While further studies are required to uncover the specific mechanisms underlying this phenomenon, a correlation between brain parenchymal volume and cognitive function was previously demonstrated (O’Sullivan et al., 2004). Cognitive dysfunction has also been associated with markers of brain atrophy in the corpus callosum (Yamauchi et al., 1994), GM, and hippocampus (Mungas et al., 2001). Together, these results suggest that WM volume is related to cognitive function, in part by reflecting brain atrophy. The existence and progression of WMH and lacunar infarction in the lateral ventricle have also been associated with the aggravation of brain atrophy but not with vascular risk factors (Kloppenborg et al., 2012). While brain atrophy plays an important role in the pathogenesis of WMH, brain atrophy and WMH often occur simultaneously. Thus, considering WMH only in the evaluation of cognitive dysfunction will likely lead to deviations. In contrast, the ratio of WMH to total WM volume may reduce the impact of brain atrophy to more accurately reflect the likelihood of cognitive dysfunction.

There are some limitations to this study. Firstly, the sample size was small due to the high incidence of CSVD. We plan to conduct further research incorporating a larger sample and improve the longitudinal aspect to further elucidate these results. Secondly, the cross-sectional study design and the combination of longitudinal studies in the study may make the results more reliable.

## 5. Conclusion

In this study, we quantitatively analyzed the WMH volume and WM volume of patients with CSVD using medical image analysis software, observing that both were correlated with cognitive impairment. Both WMH volume and WM volume were also identified as independent risk factors for cognitive impairment. However, they may exert different effects on different aspects of cognition. WMH may be related mainly to cognitive processes involving visual space, executive function, and delayed memory. Our results also indicate that the ratio of WMH to total WM volume may reduce the impact of brain atrophy, allowing for more accurate evaluation of cognitive dysfunction in older adults with CSVD. The current study ultimately provides new insight into the pathological mechanisms by which WMH is related to cognitive dysfunction.

## Data availability statement

The original contributions presented in this study are included in the article/supplementary material, further inquiries can be directed to the corresponding author.

## Ethics statement

All studies involving human participants were reviewed and approved by the Ethics Committee of the Second Affiliated Hospital of Zhengzhou University. The patients/participants provided their written informed consent to participate in this study. Written informed consent was obtained from the individual(s) for the publication of any potentially identifiable images or data included in this article.

## Author contributions

SZ was responsible for the conception and design of the research plan, the implementation and feasibility analysis of the research, data collection, collation, statistical analysis, interpretation of results, generation of figures, and manuscript writing. YH and HY were responsible for patient enrollment and baseline data collection. QL and JC were responsible for article quality control and review, supervision, and management. HB was responsible for the research program design guidance, the final version of the revision, and was responsible for the article. All authors contributed to the article and approved the submitted version.
